# Acute kidney injury and its outcomes in melioidosis

**DOI:** 10.1007/s40620-021-00970-x

**Published:** 2021-01-30

**Authors:** Ravindra Attur Prabhu, Tushar Shaw, Indu Ramachandra Rao, Vandana Kalwaje Eshwara, Shankar Prasad Nagaraju, Srinivas Vinayak Shenoy, Chiranjay Mukhopadhyay

**Affiliations:** 1 Department of Nephrology, Kasturba Medical College, Manipal, Manipal Academy of Higher Education, Manipal, Udupi, 576104 Karnataka India; 2 Department of Microbiology, Kasturba Medical College, Manipal, Manipal Academy of Higher Education, Manipal, Karnataka India

**Keywords:** Acute kidney injury, Melioidosis, Bacteraemia, Sepsis, *Burkholderia pseudomallei*

## Abstract

**Background:**

Melioidosis is a potentially fatal tropical infection caused by *Burkholderia pseudomallei*. Kidney involvement is possible, but has not been well described.

**Aim:**

This study aimed to assess the risk of acute kidney injury (AKI) and its outcomes in melioidosis.

**Methods:**

A retrospective observational cohort study was performed. Case records of consecutive patients with culture-confirmed melioidosis, observed from January 1st, 2012 through December 31st, 2019 were analysed for demographics, presence of comorbidities, including chronic kidney disease (CKD), diabetes mellitus (DM), and presence of bacteraemia, sepsis, shock, AKI, and urinary abnormalities. The outcomes we studied were: mortality, need for hospitalisation in an intensive care unit (ICU), duration of hospitalization. We then compared the outcomes between patients with and without AKI.

**Results:**

Of 164 patients, AKI was observed in 59 (35.98%), and haemodialysis was required in eight (13.56%). In the univariate analysis, AKI was associated with CKD (OR 5.83; CI 1.140–29.90, *P* = 0.03), bacteraemia (OR 8.82; CI 3.67–21.22, *P* < 0.001) and shock (OR 3.75; CI 1.63–8.65, *P* = 0.04). In the multivariate analysis, CKD (adjusted OR 10.68; 95% CI 1.66–68.77; *P* = 0.013) and bacteraemia (adjusted OR 8.22; 95% CI 3.15–21.47, *P* < 0.001) predicted AKI. AKI was associated with a greater need for ICU care (37.3% vs. 13.3%, *P* = 0.001), and mortality (32.2% vs. 5.7%, *P* < 0.001). Mortality increased with increasing AKI stage, i.e. stage 1 (OR 3.52, CI 0.9–13.7, *P* = 0.07), stage 2 (OR 6.79, CI 1.92–24, *P* = 0.002) and stage 3 (OR 17.8, CI 5.05–62.8, *P* < 0.001), however kidney function recovered in survivors. Hyponatremia was observed in 138 patients (84.15%) and isolated urinary abnormalities were seen in 31(18.9%).

**Conclusions:**

AKI is frequent in melioidosis and occurred in 35.9% of our cases. Hyponatremia is likewise common. AKI was predicted by bacteraemia and CKD, and was associated with higher mortality and need for ICU care; however kidney function recovery was observed in survivors.

**Graphic abstract:**

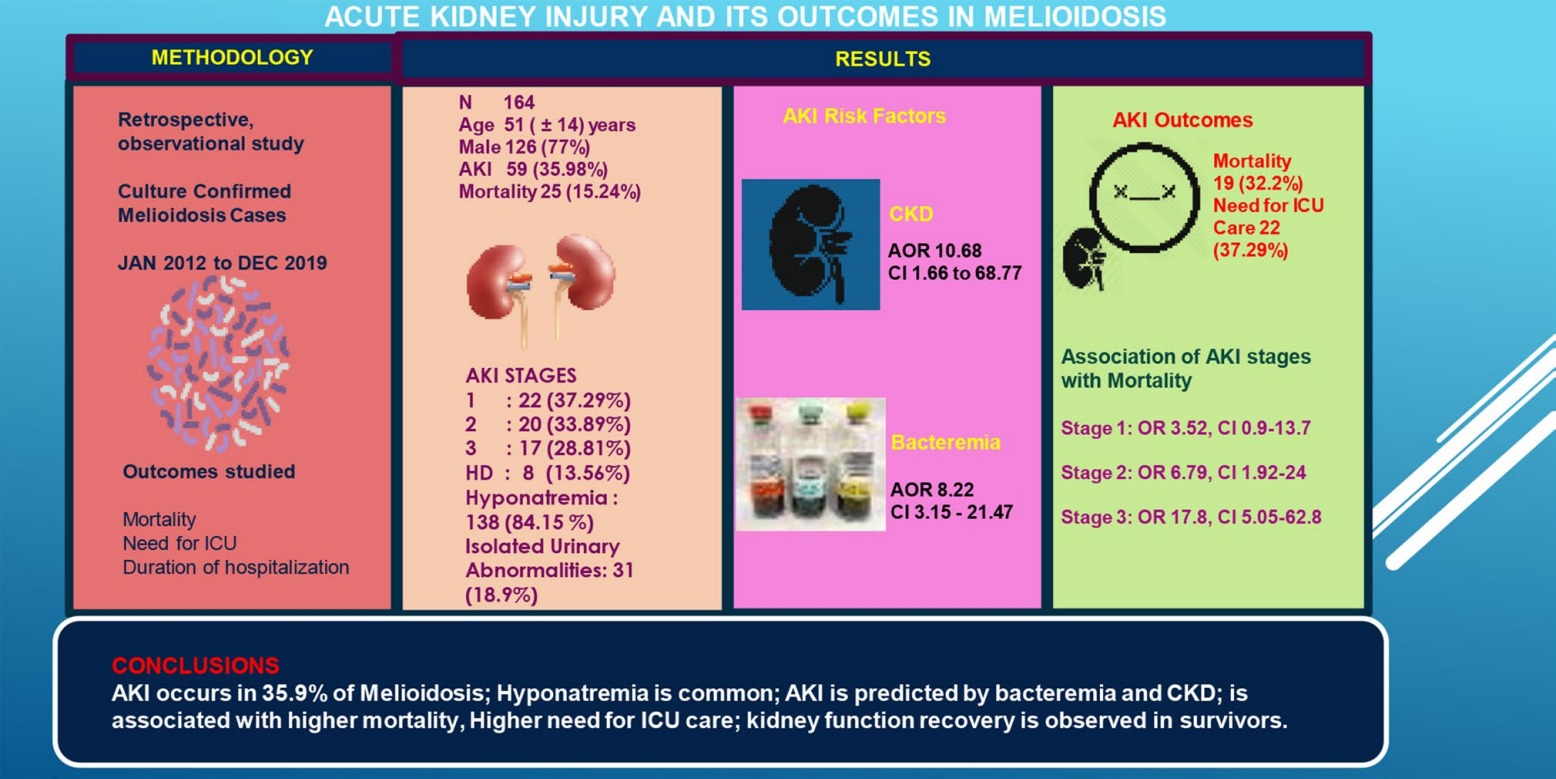

## Introduction

Melioidosis is a potentially fatal tropical disease caused by *Burkholderia pseudomallei*, a soil-dwelling gram-negative bacillus endemic to South East Asia and North Australia, that can affect travellers to these areas. It has been estimated that about 165,000 new cases occur yearly worldwide, of which 89,000 are fatal. Although largely under-reported in the Indian subcontinent, this region is believed to account for 44% of cases, with an estimated annual incidence of 52,500 cases per year in India [1]. A recent review paper from India identified a total of 583 cases of melioidosis in India between 1991 and 2018, mostly from Karnataka and Tamil Nadu states [2]. Disease transmission predominantly occurs through percutaneous inoculation and commonly manifests as pneumonia, abscess formation, sepsis, and multi-organ dysfunction. Genitourinary involvement in the form of renal and lower urinary tract abscesses have been well described [3–7]. However, very few studies have focused on the kidney manifestations of melioidosis. Although acute kidney injury (AKI) was reported in 35% of patients with melioidosis in a study conducted in 1987, few data are available concerning AKI following the introduction of standard therapy with ceftazidime and carbapenem [8]. We studied the kidney manifestations of melioidosis and their impact on patient outcomes.

## Methods

### Study design and setting

This is a single-centre, record-based analysis of the melioidosis registry of a tertiary care referral facility in the southern part of Karnataka state, India. After hospital ethics committee approval, demographic, clinical and laboratory data were collected and subjects with AKI were identified using the Serum Creatinine-based definition of the Kidney Disease Outcomes Quality Initiative/Acute Kidney Injury Network (KDOQI/AKIN) criteria [9].

### Study participants

Adults (> 18 years) with confirmed melioidosis based on a positive culture for *B. pseudomallei* from any clinical specimen between January 2012 and December 2019 were included in the study. Those with chronic kidney disease stage 5 and 5D were excluded. All patients had received intravenous antibiotics, either Ceftazidime or Carbapenem, with or without Cotrimoxazole, as per standard treatment guidelines.

### Outcomes

We studied the need for intensive care unit (ICU) care, duration of hospitalization and in-hospital mortality. The covariates we studied were age, gender, comorbidities including diabetes mellitus (DM), chronic liver disease (CLD), chronic kidney disease (CKD), and type of clinical presentation.

### Study definitions

A confirmed case of melioidosis was defined as a patient with a positive culture for *B. pseudomallei* from any clinical specimen, while bacteraemia was defined as a positive blood culture for *B. pseudomallei*. Localized melioidosis was defined as the involvement of a single organ system, disseminated melioidosis as the involvement of two or more sites with bacteraemia, and multifocal melioidosis as the involvement of two or more sites without bacteraemia. Acute presentation was defined as subjects with symptoms lasting less than 2 months at entry. KDOQI/AKIN criteria were used to diagnose and stage acute kidney injury [9]. The estimated glomerular filtration rate (eGFR) was calculated using the CKD-EPI equation [10]. Sepsis was defined as per the Sepsis-3 criteria [11]. Proteinuria was defined as urine dipstick ≥ 1 + , and microscopic haematuria as ≥ 5 RBCs/high power field at urine microscopy. Hyponatremia was defined as serum sodium < 135 meq/L, and severe hyponatremia as serum sodium < 120 meq/L.

### Statistical analysis

Statistical analysis was performed using SPSS version 22.0. Descriptive data were presented as mean and standard deviation (SD) for normally distributed continuous variables, and as median and interquartile range (IQR) for skewed variables. Data were presented as frequency (n) and percentages (%) for categorical data. Comparison of proportions was performed using the Chi-squared test and comparison of non-parametric data by the Mann–Whitney *U* test. To identify factors predictive of AKI, univariate and multivariate logistic regression analysis was performed. The confounders we considered were age, gender, diabetes mellitus, hypertension, CKD, CLD, bacteraemia and shock. Crude and adjusted odds ratios were calculated with 95% Confidence Intervals (CI). All results were considered significant at a *P*-value of < 0.05.

## Results

### Demography

Of 164 patients, 126 (77%) were males, mean age was 51 ± 14 years. Baseline characteristics are summarized in Table 1.

**Table 1:** Baseline characteristics

Characteristics	Number (*N* = 164)	AKI (*N* = 59)	Without AKI (*N* = 105)	*P*
Age, years (mean ± SD)	50.92 ± 13.83	52.02 ± 13.15	50.31 ± 14.22	0.45
Sex men/women	126/38	49/10	77/28	0.18
Co-morbidities*				
Diabetes mellitus	125 (76)	45 (76)	80 (76)	1.00
Hypertension	45 (27)	15 (25)	30 (28)	0.72
Chronic kidney disease	8 (5)	6 (10)	2 (2)	**0.03**
Chronic liver disease	7 (4)	5 (8.5)	2 (2)	0.09
Malignancy	5 (3)	0 (0)	5 (5)	0.16
Clinical presentation*				
Acute	160 (98)	59 (100)	101 (96)	0.29
Pneumonia	68 (42)	31 (53)	37 (35)	0.021
Abscess	71 (43)	13 (22)	58 (55)	**< 0.001**
Septic arthritis/osteomyelitis	33 (20)	13 (22)	20 (19)	0.687
Encephalomyelitis	4 (2.4)	2 (3.5)	2 (1.9)	0.441
Bacteraemia	100 (61)	52(88)	48 (46)	**< 0.001**
Shock	29 (18)	18 (62)	11 (38)	**0.002**
Type of involvement*				
Localized	50 (30)	5 (8)	45 (43)	**< 0.001**
Bacteraemia alone	12 (7)	5(8)	7 (7)	
Disseminated involvement	88 (54)	47 (80)	41 (39)	
Multifocal involvement	14 (9)	2 (3)	12 (11)	

Bold values indicate statistically significant *P* values (*P* < 0.05)

*Number (%)

### AKI

AKI was seen in 59/164 (35.98%) patients, with 22 (37.29%), 20 (33.89%) and 17 (28.81%) in stage 1, 2 and 3, respectively (Table 2). Eight (4.8%) required haemodialysis. Among those with AKI, urinary abnormalities such as microscopic haematuria and proteinuria were seen in 19/59 (32.2%) and 37/59 (62.7%), respectively. All survivors (38 of 59 patients) had complete renal recovery.

**Table 2:** Acute kidney injury and other kidney manifestations in melioidosis

Characteristics	Total (number = 59)
Oliguria*	11 (19)
Blood urea at admission, mmol/L^a^	7.8 (5.3, 12)
Serum creatinine at admission in µmol/L^a^	121.9 (86.6, 216.5)
Estimated glomerular filtration rate at admission, mL/min/1.73m^2^^b^	61 ± 36
Blood urea nitrogen/serum creatinine ratio > 20*	16 (27)
Nadir estimated glomerular filtration rate, mL/min/1.73m^2^^b^	39 ± 26
Proteinuria*	37 (63)
Haematuria*	19 (32)
Need for haemodialysis*	8 (14)

*Number (%)

^a^Median (interquartile range

^b^Mean (Standard deviation)

AKI was more common in patients with CKD (10.17% vs. 1.9%, *P* < 0.001), bacteraemia (88.1% vs. 45.71%, *P* < 0.001), shock (62.1% vs. 37.9%, *P* = 0.002) and disseminated involvement (79.66% vs. 39.05%), while those with localized involvement had lower rates (8.47% vs. 42.86%, *P* < 0.001).

In the univariate analysis, CKD (OR 5.83; CI 1.140–29.90, *P* = 0.03), bacteraemia (OR 8.82; CI 3.67–21.22, *P* < 0.001) and shock (OR 3.75; CI 1.63–8.65, *P* = 0.04) were associated with AKI (Table 3). CKD (adjusted OR 10.68; CI 1.66–68.77, *P* = 0.013) and bacteraemia (adjusted OR 8.22; CI 3.15–21.47, *P* < 0.001) were independently associated with AKI in multivariate analysis.

**Table 3:** Predictive factors of acute kidney injury in melioidosis

Predictive factor	Unadjusted odds ratio (95% CI)	*P* value	Adjusted odds ratio (95% CI)	*P* value
Age	1.00 (0.99–1.03)	0.45	–	
Male sex	1.78 (0.79–3.99)	0.16	–	
Diabetes mellitus	1.00 (0.48–2.13)	0.99	–	
Hypertension	0.85 (0.41–1.76)	0.67	–	
Chronic liver disease	5.20 (0.96–27.70)	0.05	–	
Chronic kidney disease	5.83 (1.140–29.90)	**0.03**	10.68 (1.66–68.77)	**0.013**
Bacteraemia	8.82 (3.67–21.22)	** < 0.001**	8.22 (3.15–21.47)	** < 0.001**
Shock	3.75 (1.63–8.65)	**0.002**	2.2 (0.90–5.37)	0.084

Subjects with AKI had significantly higher in-hospital mortality [19/59 (32.20%) vs. 6/105 (5.71%), *P* < 0.001] (Table 4) and greater need for ICU care [22/59 (37.29%) vs. 14/105 (13.34%), *P* = 0.001], but similar median number of days of hospitalization (16 vs. 13, *P* = 0.248). AKI was also independently associated with mortality (adjusted OR 7.89; CI 1.97–31.59, *P* = 0.003) in univariate and multivariate regression analysis. Mortality increased with increasing AKI stage, i.e. stage 1 (OR 3.52, CI 0.9–13.7, *P* = 0.07), Stage 2 (OR 6.79, CI 1.92–24, *P* = 0.002) and Stage 3 (OR 17.8, CI 5.05–62.8, *P* < 0.001), and was significant for stages 2 and 3.

**Table 4:** Impact of acute kidney injury on outcomes in melioidosis

Outcomes	With AKI (*N* = 59)	Without AKI (*N* = 105)	*P* value
Mortality*	19 (32)	6 (6)	< 0.001
Need for intensive care*	22 (37)	14 (13)	0.001
Hospitalization days^a^	16 (8, 23)	13(7, 19.5)	0.248

*Number (%)

^a^Median (inter quartile range)

#### Other manifestations

Other renal manifestations included urine abnormalities in 31(18.9%), (viz.) proteinuria in 18(10.98%), microscopic haematuria in 5(3.05%), or both in 8(4.88%).

Of note, hyponatremia was observed in 138 subjects (84.15%), and was severe (Na < 120 meq/L) in 26 (15.85%) of them.

#### Genitourinary involvement

Prostate abscesses were found in three individuals (1.83%).

## Discussion

Melioidosis is caused by the facultative gram-negative intracellular bacterium *Burkholderia pseudomallei* that is found in soil and in fresh surface water. It is endemic to Northern Australia, South East Asia and tropical regions and is considered to be a potential bio threat. Diabetes, CKD, chronic lung disease and alcohol abuse are risk factors. After skin inoculation, the organism spreads haematogenously, evades innate immunity using surface polysaccharides, escapes host cell autophagy by activating pattern recognition receptors and causes host cell fusion to form multinucleated giant cells with intracellular survival using proteins which inhibit oxidant systems [12]. The disease is associated with a case fatality rate of 10–50% [13, 14]. Like other tropical infections, melioidosis is associated with multiorgan dysfunction, affecting the liver, heart, and brain. However, to our knowledge, there are few studies describing kidney manifestations of melioidosis.

In a retrospective study carried out in Thailand in 1987, Susaengrat et al. studied 220 patients with melioidosis, of whom 77 (35%) developed AKI (defined as serum creatinine > 2 mg/dL and failure to improve with volume expansion) [8]. They also observed that AKI occurred in 61% of patients with a septicemic form of melioidosis. Renal histological findings were available from autopsy data for 13 of these patients, and the most common findings included acute tubular injury, interstitial nephritis, and micro abscesses. Similarly, Chierakul et al. found that renal failure occurred in about 30% of individuals with melioidosis in a randomized controlled trial comparing ceftazidime monotherapy and ceftazidime combined with Trimethoprim Sulphamethoxazole [15].

In our study, AKI occurred in 35.9% of patients, and aetiology was presumably pre-renal or acute tubular necrosis (ATN), as evidenced by complete renal recovery in survivors. Since BUN/creatinine ratio was < 20 in most patients with AKI, ATN appears to be the predominant aetiology. However, associated urinary abnormalities were present in some patients (proteinuria in 22.6% and microscopic haematuria in 11.6%) and hence, the possibility of glomerular involvement cannot be ruled out. Differentiation between glomerular and tubular proteinuria based on the quantification of urinary protein and simultaneous estimation of low molecular weight proteins and albumin using gel electrophoresis was not carried out in our study. Advanced renal dysfunction requiring dialysis was uncommon and was seen in 13.56% of individuals with AKI. Like the findings of Susaengrat et al., the presence of bacteraemia was independently associated with a higher occurrence of AKI (adjusted OR 8.22; 95% CI 3.15–21.47, *P* < 0.001) in our study.

The presence of AKI was associated with poorer outcomes. The overall mortality rate was 15.2% which was higher in patients with AKI (32.2% vs. 5.7%, *P* < 0.001). However, it was lower than the 89.6% mortality rate reported earlier by Susaengrat et al*.* [8]. This appears to be attributable to earlier detection and more effective therapies in the current era. Ceftazidime and Carbapenem therapy have halved mortality rates in melioidosis [16, 17]. In the study by Susaengrat et al*.*, patients were treated with a combination of Chloramphenicol, Doxycycline and Cotrimoxazole, whereas patients in our study received Ceftazidime or Carbapenem therapy, either alone or in combination with Cotrimoxazole, as per current practice guidelines. Those with AKI also had a greater need for ICU care indicating that kidney involvement is a marker of severe disease. AKI was also an independent predictor of mortality, consistent with previous studies [15].

Hyponatremia is a common, albeit poorly studied manifestation of melioidosis. Previous studies have reported hyponatremia in up to 90% of patients [8, 18, 19]. In our study, 84.1% had hyponatremia, with severe hyponatremia being observed in 15.9% of patients. The possibility of a melioidosis-associated syndrome of inappropriate antidiuretic hormone secretion (SIADH) has been suggested by a few authors [18, 19]. Although the exact mechanism of hyponatremia in melioidosis is unclear, its presence may be an important clinical clue for diagnosis, similar to Legionnaire’s disease [20].

We also found that isolated urinary abnormalities occurred in 18.9% of patients. In those with AKI, 11.6% had microscopic haematuria and 22.6% had dipstick proteinuria. Data on serum complement levels were not available and none of the patients had undergone kidney biopsy. Although it is unknown whether these findings represent possible glomerular involvement, some case reports have postulated that melioidosis may have glomerular involvement. Northfield et al. reported a case of melioidosis presenting with nephrotic syndrome and low complement levels, which resolved with antimicrobial therapy alone [21]. Although renal biopsy was not performed in this patient, the authors postulated that alternate complement pathway activation leading to immune-complex glomerulonephritis was the probable aetiology in this patient. Anti-Glomerular basement membrane(GBM)-mediated glomerulonephritis was reported in a patient with severe community-acquired pneumonia due to melioidosis, who also had anuric renal failure, which was subsequently proved to be due to crescentic glomerulonephritis consistent with anti-GBM disease, with serological evidence of anti-GBM antibodies and the presence of pulmonary haemorrhage [22].

**Fig. 1:**
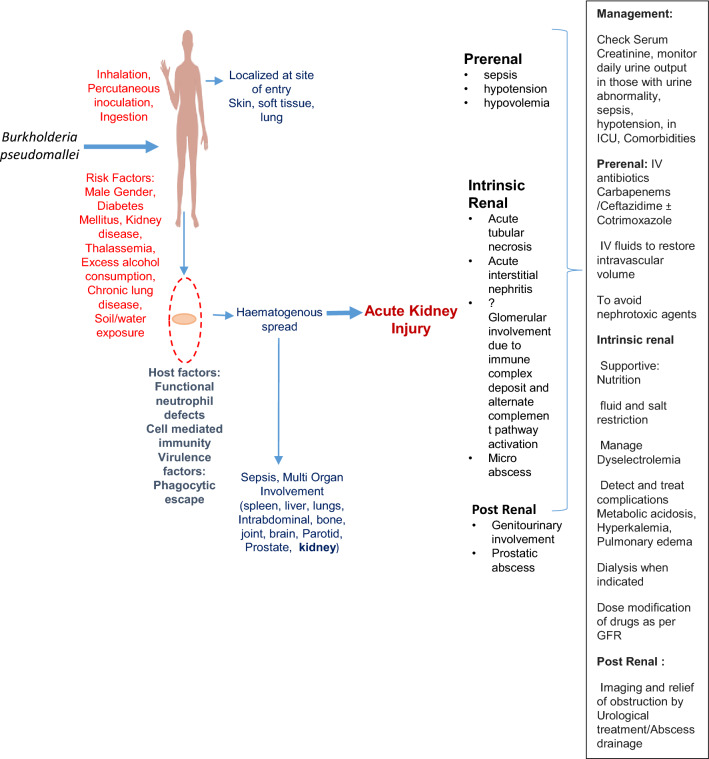
Mechanism, types of kidney involvement and management of acute kidney injury in melioidosis

While the physiopathology of AKI in melioidosis is unclear, possibilities include pre-renal causes following sepsis, hypotension, intrinsic renal disease i.e. acute tubular necrosis, interstitial nephritis or rarely, glomerular involvement, while post renal causes may be due to genitourinary and prostatic causes (Fig. 1). Management of melioidosis should include early recognition and management of AKI. Besides timely initiation of antibiotics, pre-renal AKI requires aggressive intravascular volume resuscitation in order to avoid progression to acute tubular necrosis. Management of intrinsic renal AKI is mainly supportive. Kidney biopsy may be required in the presence of persistent proteinuria and non-resolving AKI. Other therapies that have been attempted in melioidosis include drotrecogin alfa or activated protein C, which showed some benefit, and lexipafant, a platelet-activating factor receptor antagonist which, however, exhibited no effect [13]. A randomized controlled trial involving the use of granulocyte colony stimulating factor in severe sepsis due to melioidosis showed longer survival but no benefit with regard to mortality [23].

The main strength of this study is the description of AKI in a recent large cohort of patients affected by melioidosis, while a limitation of this study is the lack of renal histopathological data. Since most patients had a mild, self-resolving form of AKI, a kidney biopsy was not performed, hence the exact  pattern of AKI could not be assessed. Secondly, this being a retrospective hospital based, single-centre study, and it may not reflect the pattern of melioidosis in the community.

To conclude, in our series, AKI occurred in 35.9% of patients with melioidosis, was associated with the presence of bacteraemia and CKD, and  was associated with higher mortality (especially in stage 2 and 3 patients), need for ICU care and mechanical ventilation. Most cases of AKI were in stage 1 or 2 and complete recovery of kidney function was observed in survivors. Although acute tubular necrosis is the most likely aetiology, associated urinary abnormalities may indicate glomerular involvement in at least some patients. Hyponatremia was a common finding and was recorded in 85% of patients.

## Data Availability

The datasets generated and/or analysed during the current study are available from the corresponding author on reasonable request.
